# The prevalence of depression in pregnant patients and its associated risk factors: A cross sectional study at the obstetrics clinic of a university hospital

**DOI:** 10.12669/pjms.42.7.14762

**Published:** 2026-07

**Authors:** Esranur Akilli Odemis, Cigdem Gereklioglu

**Affiliations:** 1 Esranur Akilli Odemis Seyhan Health Directorate, Sumer Family Health Center, Adana, Turkiye; 2 Cigdem Gereklioglu Department of Family Medicine, Çukurova University, Adana, Türkiye

**Keywords:** Antenatal depression, Pregnant women, Risk factors

## Abstract

**Objectives:**

Pregnancy is a physiologic condition that leads to many metabolic, cellular, and hormonal changes. Depression is a common mental illness among women of reproductive age group, particularly during pregnancy, childbirth, and puerperium. The present study aimed to examine the prevalence of depression in pregnant women to find associated risk factors, and screening for S/S of depression.

**Methodology:**

This cross-sectional descriptive study was conducted on 311 pregnant women aged 18 years and over, with a pregnancy of 14 weeks or more, who were admitted to Cukurova University Obstetrics Outpatient Clinic between August 1, 2022, and September 15, 2022. Sociodemographic data Form, the obstetric examination Form, and the Edinburgh Postpartum Depression Scale (EPDS) were used for data collection. The statistical analysis was evaluated using SPSS (Statistical Package for the Social Sciences) 20.0 package program, and the statistical significance level was taken as p<0.05.

**Results:**

The mean age was 28,9±5,9 years; 42.1% were graduates of primary school, and 97.7% were married, 18.6% had consanguineous marriage. Of the patients, 84.9% were not working, partners of 89.1% were working, 80.1% were living in a nuclear family, and 62.7% reported that their monthly income was insufficient. There were no chronic disorders in 79.4% of the participants, 83.0% were not smoking, and 79.4% were not doing regular exercise. Based on EPDS, 33.76% were found to have a high risk for depression. Having a previous diagnosis of depression, living in an extended family, smoking, not receiving support from the partner, and having insufficient monthly income increased the depression risk levels (p=0,001; p<0,001; p=0,002; p=0,015; p=0,028, respectively)

**Conclusion:**

The risk for antenatal depression is common among women. Screening for the signs and symptoms of depression in pregnant women would contribute to the prevention of depression, improve maternal and fetal health, and also the family life.

## INTRODUCTION

Pregnancy is a physiologic condition that leads to a variety of metabolic, cellular, and hormonal changes in the females. So, it leads to difficulties in adjustment on physical, psychological, and social aspects.^1^ Depression is a mental illness in which the person loses the will to live, accompanied by a deep sadness, slowing down in thoughts and speech, and disruptions in physiological functions, and also by feelings and thoughts such as stagnation, feeling worthless, inability to enjoy life, powerlessness, and pessimism.^2^ The majority of depression is seen in women between the ages of 18 and 44, including periods such as pregnancy, childbirth, and puerperium.^3^

The ratio of depression during pregnancy varies between 15% to 65% in the World^4^ and between 27% to 54% in our country.^3^ The likelihood of peripartum complications, cesarean section and/or interventional delivery, and prolonged labor is higher among those who experience depression during pregnancy.^5^ Antepartum depression increases the risk of postpartum depression.^6^

Despite the significant adverse effects of depression on the mother and the baby, studies investigating antenatal depression are lacking.^7^ Recognizing depression during and after pregnancy, identifying risk factors, and preventing adverse outcomes is an important issue for family medicine. The present study aimed to investigate the prevalence of depression and its risk factors among pregnant women. This can lead to early recognition and thereby preventing its further complications.

## METHODOLOGY

A cross-sectional descriptive study was conducted among 311 pregnant women admitted to the Obstetrics Outpatient Clinic of Cukurova University between August 1, 2022, and September 15, 2022.

### Ethical Approval:

Ethics committee approval was obtained from the Non-interventional Research Ethics Committee prior to the study (Date: June 3, 2022; number: 42/123). Verbal and written informed consent was obtained from the participants. The study was conducted per the ethical principles of the Helsinki Declaration.

### Inclusion criteria:

- Being 18 years old or above,
- Being literate,
- In the second or third trimester of pregnancy,
- A volunteer for participation.

### Exclusion criteria:

- Being under 18 years of age,
- Having a pregnancy through assisted fertility techniques,
- Being in the first trimester,
- Having a physical and/or cognitive disorder. (The reason for allocating the patients after 14 weeks of pregnancy was that a detailed ultrasonography examination was started to be performed after 14 weeks of gestation and we needed the detailed obstetrics information for the study).

### Data collection tools:

*The sociodemographic data collection form* composed of 19 open and closed-ended questions inquiring about the sociodemographic characteristics, chronic disorders, smoking and exercise habits, pregnancy characteristics (the number of the pregnancies, outcomes of previous pregnancies, history of risky pregnancies, whether the present pregnancy is planned or not), and psychosocial features.

*The obstetric examination form* is composed of 17 questions and inquiries about past obstetric history, habits (smoking, alcohol, or substance use, medications), information about the current pregnancy, physical examination findings, and ultrasonographic measurements of fetus and uterus.

*The Edinburgh Postpartum Depression Scale (EPDS)* was developed by Cox et al. in 1987.^8^ It has 10 Likert-type questions. Each question is scored between 0 and 3. The possible minimum score is 0, and the maximum is 30. The cut-off point is 13, and women with a scale score of 13 or more were considered to be in the risk group. It is the only self-assessment scale validated for use both during and after pregnancy.^9^ The Turkish validity and reliability study was conducted by Engindeniz et al. in 1996, and the sensitivity and specificity of the Turkish version were 84% and 88%, respectively.^10^ The scale was applied by the researcher by face-to-face interview method and it took an average of 5-10 minutes.

### Statistical analysis:

IBM SPSS 20 package program (SPSS, Inc., Chicago, Illinois, USA) was used for statistical analysis of the data. Categorical measurements were summarized as number (n) and percentage (%), and continuous measurements were summarized as mean ± standard deviation, median, and minimum-maximum. The chi-square test and the Fisher’s exact test were used for comparisons of categorical variables. The Kolmogorov-Smirnov test was used to determine whether the parameters showed a normal distribution. The Mann-Whitney U test was used to compare the two groups in parameters that did not show a normal distribution. To determine the factors affecting the depression level, the variables that were significant in the single analysis were added to the multivariate logistic regression model, and the final model was created by using the Forward LR method. The odds ratio and confidence intervals obtained for each variable in the model were presented. Statistical significance level was taken as p<0.05 in all tests.

## RESULTS

The mean age of 311 participants was 28,9±5,9 years; 42.1% were graduates of primary school, and 97.7% were married. Sociodemographic characteristics of the participants are presented in Table-I. Thyroid disorders were the most common chronic disorder (n=23, 35,9%) followed by diabetes in 13 patients, pulmonary diseases in 10 patients, hypertension in 8 patients, heart diseases in seven patients, renal diseases in five patients, central nervous system disorders in five patients, rheumatic diseases in five patients, and a hematologic disorder in only one patient.

**Table-I:** Sociodemographic characteristics of the participants.

	Number (n)	Percent (%)
***Educational status***		
Barely literate	18	5,8
Primary school	131	42.1
High school	93	29.9
University	69	22.2
***Occupational status***		
Working	47	15.1
Not working	264	84.9
Marital status		
Single	7	2.3
Married	304	97.7
***Occupational status of the partner***		
Working	277	89.1
Not working	34	10.9
Monthly income*		
Sufficient	116	37.3
Not sufficient	195	62.7
***Family type***		
Nuclear	249	80.1
Extended	62	19.9
Chronic disorders **		
No	247	79,4
Yes	64	20,6
Smoking***		
Yes	39	12.5
Smoking cessation during pregnancy	14	4.5
No	258	83.0
Regular exercise****		
Yes	64	20.6
No	247	79.4
***Consanguineous marriage***		
Yes	58	18.6
No	253	81.4
	*Mean±SD*	*Median (Min-Max)*
Age	28.9±5.9	29 (18-43)

SD: Standard deviation, Min: Minimum, Max: Maximum,

* No cut-off, based on the perception of the participant).

** Thyroid disorder, diabetes mellitus, hypertension, pulmonary disease, heart disease, renal disease, CNS disorder, rheumatic disease, hematologic disorder,

*** Number of packages was not questioned,

**** Those who do any mild-t-moderate exercises like walking/pilates on at least three days of the week were accepted to do regular exercises.

### Pregnancy characteristics:

Of the pregnant women, 155 (49.8%) were in the 2nd trimester and 156 (50.2%) were in the 3rd trimester. 295 (94.9%) had singleton pregnancies, 16 (5.1%) had multiple pregnancies. The current pregnancy was planned in 225 (72.3%) and unplanned in 86 (27.7%). While 82 (26.4%) were primigravida, 229 (73.6%) had experienced a previous pregnancy. Of these, 81 (26.0%) were having their second pregnancy, and 148 (47.6%) were having their third or subsequent pregnancies. Of these 229 women who had a past obstetric history, 99 (43.2%) had experienced a risky pregnancy in their previous pregnancies. Of 311 women included in the study, 109 (35.0%) were detected to have a risky condition. Of these, 72 (66.1%) were due to fetal, 43 (39.4%) maternal, and 6 (5.5%) both fetal and maternal factors. The most common maternal risk factor was gestational diabetes (n=20, 18.3%) followed by a family history of diabetes (n=12, 11.0%), preeclampsia/gestational hypertension (n=6, 5.5%) and a family history of hypertension (n=5, 4.6%). The most common fetal risk factors were found to be Rh incompatibility (n=21, 19.3%), an increased risk in the double test (n=15, 13.8%), placenta previa (n=13, 11.9%), and an increase in the triple test (n=7, 6.4%).

### Psychosocial parameters:

Of the participants, 139 (44.7%) had a history of premenstrual syndrome and (8.4%) had a history of depression. Of the pregnant women, 267 (85.9%) received support from their spouses during pregnancy and 23 (7.4%) had a history of spousal violence during or before pregnancy. For caring babies, 101 (32.5%) planned to receive support from their families, 3 (1.0%) planned to receive support from a babysitter, 13 (4.1%) were uncertain, and 194 (62.4%) planned to care for their babies themselves.

### EPDS:

The mean score of EPDS was 9,95±6,2 (range 0-30) and 33.76% were found to have a high risk for depression.

### Factors influencing depression risk:

When evaluated with regard to the differences between sociodemographic characteristics and depression risk groups, a statistically significant difference was found between depression risk and educational status (p=0.001), occupational status (p=0.008), occupational status of the partner (p=0.034), monthly income (p<0.001), and family type (p<0.001) (Table-II). The level of depression risk was higher among those who had a chronic disorder (p=0.028) and those who smoke (<0.001), exercise was not associated with the risk of depression (p= 0.633).

**Table-II:** The relationship between sociodemographic factors and the risk for depression.

	Depression Risk Level		χ2	p
	Low (n=206)	High (n=105)		
	n(%)	n(%)		
***Educational status***				
Barely literate	6 (33.3)	12 (66.7)	17.307	0.001**
Primary school	79 (60.3)	52 (39.7)		
High school	66 (71.0)	27 (29.0)		
University	55 (79.7)	14 (20.3)		
***Occupational status***				
Working	39 (83.0)	8 (17.0)	6.939	0.008**
Not working	167 (63.3)	97 (36.7)		
***Marital status***				
Single	6 (85.7)	1 (14.3)	1.215	0.430
Married	200 (65.8)	104 (34.2)		
***Occupational status of the partner***				
Working	189 (68.2)	88 (31.8)	4.501	0.034*
Not working	17 (50.0)	17 (50.0)		
***Monthly income***				
Sufficient	91 (78.4)	25 (21.6)	12.334	<0.001**
Insufficient	115 (59.0)	80 (41.0)		
***Family type***				
Nuclear	179 (71.9)	70 (28.1)	17.827	<0.001**
Extended	27 (43.5)	35 (56.5)		
Consanguineous marriage				
Yes	37 (63.8)	21 (36.8)	0.191	0.662
No	169 (66.8)	84 (33.2)		

* p<0,05,

** p<0,01, χ2: chi-square.

When the relationship between the pregnancy characteristics and the level of depression risk was analyzed, the risk of depression was lower in those with a planned pregnancy (p<0.001). A statistically significant difference was not found between depression risk and the number of the pregnancies (p=0.481), presence of a past obstetric history (p=0.465), trimester (p=0.129), singleton/multiple pregnancy (p=0.386).

While the risk of depression was found to be lower in those who did not experience risk in their previous pregnancy (p<0.05), a statistically significant difference was not found between depression risk and having a risk in their current pregnancy (p=0.119).

About psychosocial parameters, depression risk was lower among those who did not have a history of premenstrual syndrome (p=0.004), who did not have a previous diagnosis of depression (p<0,001), and who were receiving support from their partner during pregnancy (p<0.001). However, no statistically significant difference was found between the level of depression and partner violence and the source of support for caring for the baby (p=0.572 and p=0.711, respectively).

The logistic regression analysis has revealed that having a previous diagnosis of depression, living in an extended family, smoking, not receiving support from the partner during pregnancy, and having insufficient monthly income increased the risk of depression (p=0,001; p<0,001; p=0,002; p=0,015; p=0,028, respectively) (Table-III).

**Table-III:** Analysis of the factors that increase the level of depression in participants.

	p	OR	%95 CI	
			Lower limit	Higher limit
Insufficient monthly income	0.028	2.19	1.09	4.41
Smoking (being a non-smoker is the reference)	0.007	Ref.	-	-
Smoking cessation during pregnancy	0.553	1.58	0.35	7.16
Still smoking	0.002	4.29	1.74	10.59
Living in an extended family	<0.001	4.37	2.00	9.54
A previous diagnosis of depression	0.001	7.21	2.27	22.89
Not receiving support from the partner during pregnancy	0.015	2.94	1.23	7.02

*p<0,05, **p<0,01, Multi-variable logistic regression test, OR: Odds ratio, CI: Confidence interval.

## DISCUSSION

Our study has revealed a high depression risk in 33.76% of pregnant women. In studies conducted by using EPDS, this ratio was found to be between 16% and 65% in studies from different regions of the World,^11^,^12^ and between 27.5% and 33.1% in studies from different regions of our country.^13^,^14^

Of our study group, 84.9% were not working, and the partners of 10.9% of the participants were not working. Depression was higher among those who were not working,^15^ and in those whose partners were not working, consistent with the literature.^12^ Of the participants, 62.7% were not satisfied with their income level, and their depression risk was higher, similar to the literature.^15^ Also, depression risk was found to decrease as the level of education increased, consistent with the literature.^11^ A good education level and economic level can reduce depression risk by increasing women’s self-esteem and providing freedom.

In our study, 19.9% of the participants were living with an extended family and the extended family type was found to increase depression risk 4.37-fold. While some study results are consistent with ours,^16^ some others have found the opposite.^14^ Turkish families have a complex structure, like those in European countries (core family type) and Eastern countries (extended family type). As the extended family type is rare in the West, literature data are limited. Although, the extended family type is common in Eastern communities, this has not been addressed as a risk factor in the studies. Smokers and ex-smokers were found to have a higher depression risk as compared to non-smokers, consistent with the literature.^17^

Depression risk was higher in those with chronic disorders. Similar to our results, a study from Brazil has found a higher depression risk among those with chronic disorders.^11^ In the literature, correlations were found between depression risk and obstetric features.^11^,^16^-^18^ Our study did not find a significant association between depression risk and obstetric features, including period of gestation, and parity index. Thompson et al. have found similar results.^19^ Depression was found to be higher in multiple pregnancies.^20^ The reason for the higher risk of depression in nulliparous women may be the fact that the woman will experience an important life event for the first time. On the other hand, some studies have reported the opposite. This may be because, in addition to the burden of present pregnancy, female has to take care of the second and third child, and the financial and moral burdens may increase. In addition to the number of pregnancies, having a miscarriage or stillbirth in previous pregnancies is a negative life experience for the mother, and it can be thought that the mother’s anxiety of experiencing the same situations again may pose a risk for depression. Our study has found a higher risk among those with negative experiences. Also, some studies from the literature have reported similar results.^5^

Our study has also found a higher risk of depression in unplanned pregnancies, similar to studies in the literature.^5^,^16^,^19^ It can be considered that the woman’s lack of financial, psychological, and social readiness for a life-changing situation such as pregnancy significantly increases the risk of depression. At this point, primary care physicians must play an active role in educating and informing their patients about modern family planning and preventing unplanned pregnancies.

The ratio of premenstrual syndrome was 44.7% in our study, and it was associated with a higher depression risk, consistent with the literature.^21^ Women, whose mental health is adversely affected by the hormonal changes of the menstrual cycle, may be more at risk for depression during pregnancy, when there are a lot of hormonal changes.

Of the participants, 8.4% had a past history of depression, and the risk of depression during pregnancy was 7.2-fold greater in this group. This ratio was found to be 22.4 in Finland.^17^ These results may be due to the triggering of a pre-existing mental disorder by the physical changes during pregnancy as well as lifestyle changes and emotional changes due to the effect of hormones, also pregnant women with a pre-existing psychiatric disorder who regularly use medication may interrupt their treatment at the beginning of pregnancy due to fear that the medication may have teratogenic effects on the fetus. Consistent with the literature, women who received support from their partners had a lower depression risk.^22^,^23^

## CONCLUSION

The risk of depression is common among pregnant women. Despite the high number of studies investigating postpartum depression, studies are lacking about antepartum depression. We suggest that our study has contributed to the literature by performing such research. In accordance with the core compatibilities of family medicine, the patients must be addressed as a whole (hollistic approach), both acute and chronic problems should be handled together (comprehensive care), and during the longitudinal follow-ups, physicians should be aware of the risk of intrapartum depression in a patient with a history of depression or premenstrual syndrome (patient-centered care). As a family physician is responsible for the health of the community (community orientation), screening for the signs and symptoms of depression in pregnant women during pregnancy would contribute to the prevention of depression, improve the maternal and fetal health, and also the family life.
